# Cerebral Small Vessel Disease Is Associated With Smaller Brain Volumes in Adults With Type 1 Diabetes

**DOI:** 10.1155/2024/5525213

**Published:** 2024-07-02

**Authors:** Tor-björn Claesson, Jukka Putaala, Sara Shams, Eero Salli, Daniel Gordin, Stefan Mutter, Turgut Tatlisumak, Per-Henrik Groop, Juha Martola, Lena M. Thorn

**Affiliations:** ^1^ Department of Radiology/HUS Medical Imaging Centre University of Helsinki and Helsinki University Hospital, Helsinki, Finland; ^2^ Folkhälsan Institute of Genetics Folkhälsan Research Center, Helsinki, Finland; ^3^ Department of Nephrology University of Helsinki and Helsinki University Hospital, Helsinki, Finland; ^4^ Research Program for Clinical and Molecular Metabolism Faculty of Medicine University of Helsinki, Helsinki, Finland; ^5^ Department of Neurology Helsinki University Hospital and University of Helsinki, Helsinki, Finland; ^6^ Department of Radiology Karolinska University Hospital, Stockholm, Sweden; ^7^ Department of Clinical Neuroscience Karolinska Institute, Stockholm, Sweden; ^8^ Department of Radiology Stanford University, Stanford, California, USA; ^9^ Joslin Diabetes Center Harvard Medical School, Boston, Massachusetts, USA; ^10^ Department of Clinical Neuroscience/Neurology Institute of Neuroscience and Physiology Sahlgrenska Academy at University of Gothenburg, Gothenburg, Sweden; ^11^ Department of Neurology Sahlgrenska University Hospital, Gothenburg, Sweden; ^12^ Department of Diabetes Central Clinical School Monash University, Melbourne, Australia; ^13^ Department of General Practice and Primary Health Care University of Helsinki and Helsinki University Hospital, Helsinki, Finland

**Keywords:** cerebral small vessel disease, magnetic resonance, type 1 diabetes, volumetry

## Abstract

**Introduction:** Type 1 diabetes has been linked to brain volume reductions as well as to cerebral small vessel disease (cSVD). This study concerns the relationship between normalized brain volumes (volume fractions) and cSVD, which has not been examined previously.

**Methods:** We subjected brain magnetic resonance imaging studies of 187 adults of both sexes with Type 1 diabetes and 30 matched controls to volumetry and neuroradiological interpretation.

**Results:** Participants with Type 1 diabetes had smaller thalami compared to controls without diabetes (*p* = 0.034). In subgroup analysis of the Type 1 diabetes group, having any sign of cSVD was associated with smaller cortical (*p* = 0.031) and deep gray matter volume fractions (*p* = 0.029), but a larger white matter volume fraction (*p* = 0.048). After correcting for age, the smaller putamen volume remained significant.

**Conclusions:** We found smaller thalamus volume fractions in individuals with Type 1 diabetes as compared to those without diabetes, as well as reductions in brain volume fractions related to signs of cSVD in individuals with Type 1 diabetes.

## 1. Introduction

Adults of different ages with Type 1 diabetes demonstrate reductions in brain volumes; however, the specific anatomic structures where brain volume reductions were most profound have varied across studies [1–5]. Furthermore, early onset of Type 1 diabetes has been shown to reduce brain growth [6, 7].

In a recent study from the Diabetes Control and Complications Trial/Epidemiology of Diabetes Interventions and Complications (DCCT/EDIC Study) [8], brain volumes were assessed in a large cohort of more than 400 middle-aged and older adults with Type 1 diabetes and 99 controls. In that study, Type 1 diabetes was associated with smaller total brain volume as well as white and gray matter volumes, equaling 4–9 years of brain aging compared to healthy controls. Also, increasing age, higher systolic blood pressure, and worse proliferative diabetic retinopathy were associated with larger white matter volume. Higher BMI, higher pulse rate, and worse peripheral diabetic neuropathy were associated with larger gray matter volumes, while higher age, higher HbA1c, and higher diastolic blood pressure were associated with smaller gray matter volumes.

Manifestations of cerebral small vessel disease (cSVD), such as lacunae, white matter hyperintensities, cerebral microbleeds, and cortical superficial siderosis, can be assessed from routine brain MRI examinations [9]. Diabetes is known to affect the brain microvasculature, with an increased prevalence of both microbleeds [10] and white matter hyperintensities [11]. Furthermore, cerebral microbleeds have been linked to retinopathy in multiple studies, indicating a link between cSVD and other manifestations of small vessel disease [12, 13]. Cerebral volumes have been linked to diabetic microvascular complications, such as retinopathy and peripheral neuropathy, in smaller cohort studies [14, 15].

Smaller brain volume [16], as well as increased cSVD [17], has also been linked to type 2 diabetes [18], and progression of cSVD has been shown to correlate with (especially gray matter) brain atrophy in a lacunar syndrome population [19]. Furthermore, higher levels of advanced glycation end products have been linked to increased risks of dementia and smaller brain volumes in a geriatric population [20]. cSVD is usually a phenomenon related to aging. In Type 1 diabetes, cSVD occurs already at a relatively young age [10]. This is probably not only because of diabetes per se, since our previous report showed no association between long-term or short-term glycemic control or glycemic variability and the presence of cSVD [21]. The driving factors for cSVD in Type 1 diabetes still need to be identified, and the relationship between signs of cSVD and cerebral volumes has not been studied in Type 1 diabetes. cSVD seems to be a more prominent feature of cerebrovascular disease in Type 1 diabetes compared to type 2 diabetes and nondiabetes. In individuals with overt stroke, a microvascular etiology is present in 61% of those with Type 1 diabetes, 42% of those with type 2 diabetes, and only 10% of those without diabetes [22].

We hypothesized that adults with Type 1 diabetes would have smaller cerebral volumes compared to those without diabetes and that diabetes-related traits, such as metabolic disturbances and cSVD, would be associated with cerebral volume reductions. Thus, we aimed to compare cerebral white and gray matter volumes on MRI in adults with Type 1 diabetes to healthy controls, and the relationships between those volumes and diabetes-related factors.

## 2. Materials and Methods

### 2.1. Study Population

The nationwide Finnish Diabetic Nephropathy (FinnDiane) Study was launched in 1997 and aims to identify risk factors for micro- and macrovascular complications in individuals with Type 1 diabetes [23]. Between 2011 and 2017, we enrolled 191 FinnDiane participants aged 18–50 years with Type 1 diabetes attending the Helsinki University Hospital study center. We were able to recruit 30 control participants before the MRI scanner was decommissioned. The control participants did not have diabetes or first-degree relatives with diabetes and were selected per sex to match the age distribution of the Type 1 diabetes group.

Type 1 diabetes was defined as disease onset before 40 years of age and the start of insulin treatment within 1 year of diagnosis. Exclusion criteria were kidney replacement therapy or known cerebrovascular disease, verified by the validated Questionnaire for Verifying Stroke-Free Status [24]. We further excluded two participants with MRI findings of multiple sclerosis, one with a history of brain surgery, and one with cerebral contusions, leaving 187 participants with Type 1 diabetes and 30 healthy controls for analysis.

All participants gave their written informed consent. The Ethics Committee of the Helsinki and Uusimaa Hospital District approved the study (HUS/3313/2018 and HUS/2184/2017), and it was conducted in accordance with the Helsinki Declaration. The study protocol has been described in detail previously [10, 23].

All participants underwent brain MRI at the Helsinki Medical Imaging Center of the Helsinki University Hospital. All examinations were performed using the same 3T scanner (Achieva, Philips, Best, The Netherlands), with a standardized protocol (acquisition parameters appear in Table S1).

All participants underwent clinical examination, within 1 year of the brain MRI, at the FinnDiane study center at the Helsinki University Hospital. The visit included measurements of height, weight, waist and hip circumferences, and systolic and diastolic blood pressure. A thorough diabetes history was taken, including the presence of chronic complications and current medication.

Blood analyses were performed for HbA1c, lipids, and lipoproteins, as well as for creatinine. Insulin sensitivity was estimated with the estimated glucose disposal rate (eGDR) formula [23, 25]. We defined albuminuria as a urinary albumin excretion rate ≥ 30 mg/24 h or ≥ 20 *μ*g/min in two out of three 24-h or overnight urine collections. We defined retinopathy as a history of retinal photocoagulation [12], coronary heart disease as a history of myocardial infarction or coronary revascularization, and peripheral vascular disease as a history of peripheral revascularization or amputation.

An experienced neuroradiologist (J.M.) reviewed all images for signs of cSVD according to the Standards for Reporting Vascular Changes on Neuroimaging (STRIVE) criteria [9], assessing microbleeds, white matter hyperintensities, and lacunae [9, 10, 26]. cSVD was defined as the presence of any of these signs. For white matter hyperintensities, having more than one hyperintense lesion was considered a significant burden. Cerebral microbleeds were further categorized as zero, one to two, or three or more because having > 2 microbleeds was previously linked to more retinopathy and a smaller callosal cross-sectional area in our cohort [12, 27].

#### 2.1.1. Brain Volumetric Analysis

Volumetric analysis was performed using the FreeSurfer 6.0 software (http://surfer.nmr.mgh.harvard.edu/) [28]. The pial surface and gray–white matter boundaries were manually inspected by a radiology fellow (T.C.), adjusted, and rerun until the segmentation matched the MRI image on standard window settings at a clinical grade workstation and environment. We compensated for the known bug where FreeSurfer 6.0 reports volumes as a number of voxels instead of in cubic millimeters (https://surfer.nmr.mgh.harvard.edu/fswiki/BrainVolStatsFixed).

We analyzed volumes of cerebral cortical and white matter, as well as those of deep gray matter structures, including the thalamus, amygdala, nucleus accumbens, nucleus caudatus, putamen, pallidum, and hippocampus. We also measured the total intracranial volume [29]. Intracranial volume increases as the brain grows but remains constant after maturation has completed, with cranial sutures closing in the early third decade [30, 31]. This property of the intracranial volume, that it does not change after being established at the end of maturation, means that the intracranial volume can be used as a reference for judging atrophy. By dividing the volumes of different parts of the brain by intracranial volume, we achieve a measure that is corrected for individual differences in brain size (including those caused by sex) and shrinks with atrophy [32]. Such normalized measures are referred to as volume fractions.

#### 2.1.2. Statistical Analyses

Statistical analyses were performed with R (http://www.r-project.org) version 4.2.2 [33], with a two-sided *p* < 0.05 used for statistical significance.

Normality of distribution was judged by inspection of QQ-plots and histograms. For normally distributed continuous variables, we reported the mean and standard deviation. For nonnormally distributed continuous variables, we reported the median and interquartile range (IQR). Normally distributed variables were compared using Student's *t*-test. Variables of nonnormal distribution were compared using Mann–Whitney's test. Categorical variables were compared with Fisher's exact test and presented as *n* (percentage).

Differences in volume fractions between participants with Type 1 diabetes and controls were examined using Mann–Whitney's test.

Volume fractions were further examined in the diabetes group as dependent variables in linear regression models. For each such model, in addition to always including age and sex, we chose independent clinical variables based on the conditions that they should be different between the Type 1 diabetes and control groups and also significant at *p* < 0.1 in univariate analysis when used as the sole independent variable in a model predicting the volume fraction, for which we were selecting independent variables. The final models were built using AIC, optimizing backward elimination. To fulfill the linear regression assumptions, HbA1c and LDL cholesterol were modeled using their logarithms.

For the cortical volume fraction model, we excluded three outliers. For the white matter volume fraction model, we excluded two outliers. The diagnostic plots of the models are representative of real world data and included as supplemental material (Figures S1, S2, and S3).

We used logistic regression in the Type 1 diabetes group to discern independent effects, with any sign of cSVD as a dependent variable, and for independent variables, *z*-scores ($z=\left(x-\overset{¯}{x}\right)/\text{SD}$) of volume fractions (in order to achieve reasonable orders of magnitude for odds ratios) different between those with and without any signs of cSVD.

## 3. Results

The clinical characteristics of the 187 participants with Type 1 diabetes and 30 healthy controls are presented in Table 1. Age and sex distributions were well balanced. Participants with Type 1 diabetes had a higher BMI, systolic blood pressure, HbA1c, and higher prevalence of antihypertensive medication, as well as more albuminuria and retinopathy. Statin therapy was more common in participants with Type 1 diabetes, and LDL concentrations were lower. Coronary heart disease was observed only in one participant with Type 1 diabetes, and no participant had peripheral vascular disease. cSVD was more common in participants with Type 1 diabetes, especially cerebral microbleeds.

### 3.1. Cerebral Volume Fractions in Type 1 Diabetes and Healthy Controls

Cerebral volume fractions for the Type 1 diabetes and control groups are presented in Table 2. The only significant difference was a smaller thalamus volume fraction in those with Type 1 diabetes.

### 3.2. Volume Fractions in Relation to cSVD

Any sign of cSVD was associated with larger white matter volume fractions and smaller cortical, thalamus, nucleus caudatus, and putamen volume fractions (Table 3).

When these volume fractions were examined as independent variables in a logistic regression model with any sign of cSVD as an outcome, smaller caudate (OR 0.678, [95% CI 0.484, 0.930], *p* = 0.019) and putamen (OR 0.607, [95% CI 0.422, 0.844], *p* = 0.004) volume fractions remained independently associated with cSVD. After further adjustment for age, only a smaller putamen volume fraction remained borderline significant with an OR of 0.699 ([95% CI 0.481, 0.987], *p* = 0.050).

### 3.3. Brain Volumes and Clinical Factors, Subgroup Analysis for Type 1 Diabetes

Female sex was associated with larger cortical, white matter, and deep gray matter volume fractions. Older age was associated with smaller cortical but larger white matter volume fractions. Higher BMI and an absence of cerebral microbleeds were associated with larger deep gray matter volume fractions. In contrast, higher LDL cholesterol concentrations or having more than one white matter hyperintensity were associated with smaller deep gray matter volume fractions (Table 4).

## 4. Discussion

In our study, we compared cerebral volume fractions in adults with Type 1 diabetes and healthy controls. The thalamus volume fraction was approximately 6% smaller in the diabetes group. Furthermore, within the Type 1 diabetes group, we observed an association between cortical, cerebral white matter, thalamus, nucleus caudatus, and putamen volume fractions and cSVD. Higher LDL cholesterol, having more than one white matter hyperintensity, and the presence of cerebral microbleeds were associated with smaller deep gray matter volume fractions.

Thalamus volumes in individuals with Type 1 diabetes compared to controls have been described previously. Differences ranging from 5% to 7% [1, 2] and a *z*-score of 4.46 [34] have been reported. This is the same order of magnitude as our findings.

Duinkerken et al. found a trend towards smaller putamen and thalamus in those with proliferative retinopathy [35]. Another recent study with smaller thalamus volumes in Type 1 diabetes compared to controls investigated cerebral perfusion by arterial spin labeling, with 7% lower thalamus perfusion in participants with Type 1 diabetes [36]. Although these differences were not statistically significant after correction for multiple testing, they hint towards the possibility of microvascular disease explaining part of the variability in these deep gray matter volumes. Alternative explanations would include other glucose-related factors and possibly whether highly connected structures such as the thalami might be more sensitive to diffuse neurodegenerative processes, which, in addition to direct damage, could cause thalamic atrophy since damage elsewhere leads to a loss of incoming stimulus.

When relating cortical and deep gray matter volume fractions to the presence of any sign of cSVD, an association between putamen volume fraction and cSVD remained borderline significant after correcting for age. Unexpectedly, we found larger white matter volume fractions with any sign of cSVD and also with increasing age. This is a confusing finding, and similar results were reported by DCCT/EDIC [8]. We found no association between HbA1c and volume fractions, which is in line with a previous study, in the same cohort, failing to demonstrate a connection between HbA1c values measured over a period of 10 years and cSVD [21]. The optimal way to treat cSVD, or prevent its complications, is not fully known. The current best evidence is for rigorous blood pressure control, but promising new treatment options, such as DL-3-n-butylphthalide, are under investigation [37]. In our cohort, blood pressure was fairly well controlled, but our previous report indicates a role of blood pressure dysregulation in cSVD in Type 1 diabetes, with especially nocturnal blood pressure being associated with cSVD [38].

In our data, the mean total cerebral white and gray matter volumes, as well as total intracranial volume, were 2%–5% smaller in the diabetes group, but this difference was not statistically significant. This finding is in accordance with observations from other cohorts [5, 15]. In contrast, a Dutch study found that both white and gray matter volumes were significantly smaller (about 3%) in participants with Type 1 diabetes compared to controls. Their participants were slightly older than ours, with a mean age of 44 years [39]. Furthermore, a US study found participants with Type 1 diabetes (mean age 50 years) to have about 5% smaller total brain gray matter volumes [40]. A recent large study of brain volumes found absolute differences in group means of about 2% [8]. Thus, if there are brain volume differences between adults with Type 1 diabetes and healthy controls, they should probably lie in the 2%–7% range.

Our study has strengths and limitations. The main limitation of this study is the small number of control participants. We were probably underpowered to detect the differences in white matter and cortical volumes reported by the DCCT/EDIC study [8]. Moreover, we had no information on glucose levels at the time of imaging, which might have affected our signal-to-noise ratios. Strengths of the study include our well-characterized cohort of patients with Type 1 diabetes and healthy controls, as well as thoroughly reviewed MRI data regarding cSVD.

## 5. Conclusions

We found that focal changes suggestive of thalamic atrophy were related to Type 1 diabetes and that having signs of cSVD was associated with smaller deep gray matter volume fractions in the Type 1 diabetes group. Understanding the implications of these findings requires elucidating their pathophysiology, which deserves further work.

## Figures and Tables

**Table 1:** Clinical characteristics of participants with Type 1 diabetes compared to healthy controls.

	**Controls (** **N** = 30**)**	**Type 1 diabetes (** **N** = 187**)**	**p**
Sex (female)	17 (57%)	100 (53%)	0.745
Age (years)	38.4 (32.2, 42.9)	39.8 (33.0, 44.8)	0.515
Disease duration (years)	NA	21.7 (18.3, 30.3)	NA
Age at diabetes onset (years)	NA	13.5 (7.9, 22.5)	NA
C-peptide (nmol/L)	NA	0.010 (0.001, 0.030)	NA
Body mass index (kg/m^2^)	24.5 ± 3.2	26.7 ± 4.2	0.002
Systolic blood pressure (mmHg)	121 ± 11	130 ± 15	< 0.001
Diastolic blood pressure (mmHg)	76 (74, 85)	76 (71, 82)	0.339
HbA1c (%)	5.14 ± 0.23	8.15 ± 1.13	< 0.001
HbA1c (mmol/mol)	32.5 ± 2.3	65.6 ± 12.3	< 0.001
eGDR (mg/kg/min)	11.00 (10.18, 11.64)	7.56 (5.08, 9.46)	< 0.001
Creatinine (*μ*mol/L)	74 (68, 81)	68 (61, 79)	0.057
Total cholesterol (mmol/L)	4.60 (4.20, 5.40)	4.40 (4.00, 4.96)	0.200
LDL cholesterol (mmol/L)	2.62 (2.33, 3.29)	2.37 (2.00, 2.89)	0.018
HDL cholesterol (mmol/L)	1.46 (1.28, 1.67)	1.51 (1.25, 1.80)	0.328
Triglycerides (mmol/L)	0.84 (0.70, 1.24)	0.90 (0.67, 1.34)	0.420
Antihypertensive medication	0 (0%)	65 (35%)	< 0.001
Statin therapy	0 (0%)	40 (21%)	0.005
Retinal photocoagulation	0 (0%)	45 (24%)	0.003
Coronary heart disease	0 (0%)	1 (0.5%)	> 0.999
Current smoking	5 (17%)	15 (8.0%)	0.166
Intracranial volume (L)	1.67 (1.52, 1.76)	1.62 (1.49, 1.74)	0.504
Any sign of cerebral small vessel disease	3 (10%)	66 (35%)	0.006
Any cerebral microbleeds	1 (3.3%)	44 (24%)	0.011
Any white matter hyperintensities	2 (6.7%)	44 (24%)	0.036
Any lacunar infarction	0 (0%)	4 (2.1%)	> 0.999

Dichotomous factors are presented as number and percentage, variables of normal distribution are presented as mean ± sd, and variables not of normal distribution are presented as median and IQR.

**Table 2:** Cerebral volume fractions for Type 1 diabetes and controls groups, given as median and IQR.

	**Controls (** **N** = 30**)**	**Type 1 diabetes group (** **N** = 187**)**	**p**
Cortex	0.297 (0.294–0.312)	0.301 (0.291–0.316)	0.921
White matter	0.293 (0.274–0.3)	0.29 (0.276–0.302)	0.770
Deep gray matter	0.0301 (0.0296–0.0316)	0.0302 (0.0289–0.0314)	0.360
Thalamus	0.00973 (0.00923–0.0103)	0.00948 (0.00895–0.00991)	0.034
Amygdala	0.0022 (0.00214–0.00233)	0.00222 (0.00209–0.0024)	0.743
Accumbens area	0.000559 (0.000514–0.0006)	0.000561 (0.000504–0.000634)	0.660
Caudatus	0.00432 (0.00402–0.00472)	0.00431 (0.00399–0.00462)	0.468
Putamen	0.006 (0.0056–0.00637)	0.00583 (0.00553–0.00623)	0.325
Pallidum	0.00251 (0.00229–0.00259)	0.00244 (0.0023–0.00261)	0.542
Hippocampus	0.00531 (0.00503–0.00552)	0.00528 (0.00498–0.00566)	0.732

**Table 3:** Volume fractions by the presence of cerebral small vessel disease.

	**No sign of cerebral small vessel disease (** **N** = 121**)**	**Any sign of cerebral small vessel disease (** **N** = 66**)**	**p**
Cortex	0.304 (0.292–0.318)	0.297 (0.29–0.308)	0.031
White matter	0.287 (0.273–0.301)	0.293 (0.281–0.304)	0.048
Deep gray matter	0.0303 (0.0292–0.0314)	0.0298 (0.0282–0.031)	0.029
Thalamus	0.00955 (0.00902–0.00992)	0.00921 (0.00878–0.00985)	0.049
Amygdala	0.00224 (0.0021–0.0024)	0.0022 (0.00209–0.0024)	0.488
Accumbens area	0.000572 (0.00051–0.000635)	0.000543 (0.000484–0.000624)	0.238
Caudatus	0.00436 (0.00401–0.00466)	0.00421 (0.00394–0.00447)	0.028
Putamen	0.00589 (0.00564–0.00631)	0.00571 (0.00547–0.006)	0.009
Pallidum	0.00245 (0.00234–0.00264)	0.00241 (0.00223–0.00255)	0.053
Hippocampus	0.0053 (0.005–0.00567)	0.0052 (0.00495–0.00561)	0.461

**Table 4:** Results of linear regression.

	**Cortical volume fraction**	**White matter volume fraction**	**Deep gray matter volume fraction**
Age (years)	< −0.001, *p* < 0.001	< 0.001, *p* = 0.039	
Sex (women vs. men)	0.011, *p* < 0.001	0.006, *p* = 0.037	0.001, *p* < 0.001
BMI (kg/m^2^)		< 0.001, *p* = 0.033	< 0.001, *p* = 0.012
HbA1c (mmol/mol)			
log (LDL, mmol/L)			−0.002, *p* < 0.001
White matter hyperintensities, significant burden (yes vs. no)			< −0.001, *p* = 0.049
No microbleeds (yes vs. no)			0.001, *p* = 0.01

## Data Availability

The datasets generated and/or analyzed during the current study are confidential and not publicly available, but are available from the corresponding authors on reasonable request.
