# Supplementary material for: Cerebral Small Vessel Disease Is Associated With Smaller Brain Volumes in Adults With Type 1 Diabetes
Source: J Diabetes Res. 2024 Jul 2;2024:5525213. doi: 10.1155/2024/5525213 (PMC11233188; doi:10.1155/2024/5525213)

### Supplemental table 1: MRI sequence parameters.

| Sequence | TR | TE | Flip angle | Echos | Voxel size |
| --- | --- | --- | --- | --- | --- |
| T1 MPRAGE | shortest | 4.6 ms | 8° | 1 | 0.88x0.88x0.88 mm |
| 3D TOF | 25 ms | 3.5 ms | 20° | 1 | 0.28x0.54x0.50 mm |
| 3D SSH | shortest | 46 ms | 90° | 1 | 2.00x2.00 mm |
| T1 IR TSE | 2000 ms | 10 ms | (refocus 120°) | 1 | 0.98x1.22 mm |
| T2 FFE | shortest | 16 ms | 18 | 1 | 0.90x1.12 mm |
| T2 FLAIR | 11000 ms | 125 ms | (refocus 120°) | 1 | 0.65x0.87 mm |
| T2 TSE | 4000 ms | 80 ms | 90° | 1 | 0.60x0.75 mm |
| VEN BOLD | shortest | shortest | 10° | 1 | 1.00x0.99x0.50 mm |

### Supplemental figure 1: Diagnostics for deep gray matter regression


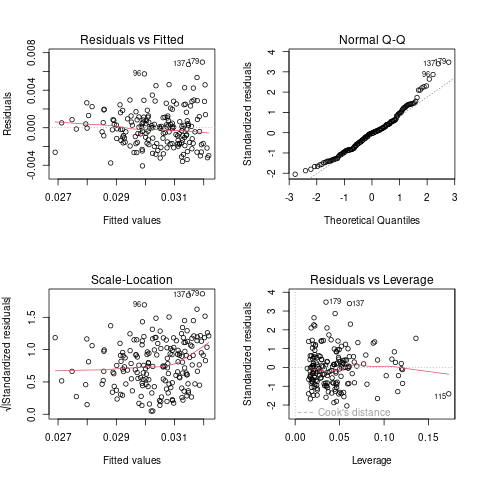


### Supplemental figure 2: DIagnostics for cortex regression

###
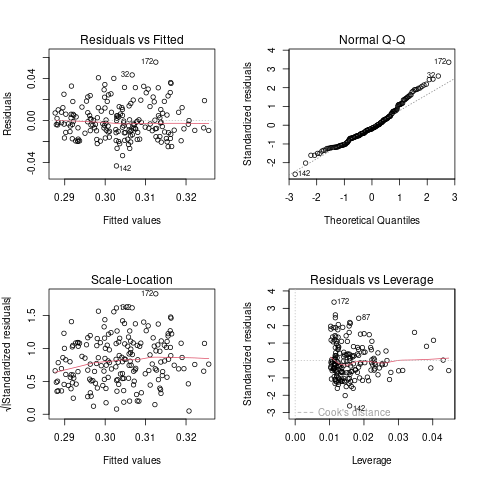


### Supplemental figure 3: Diagnostics for white matter regression
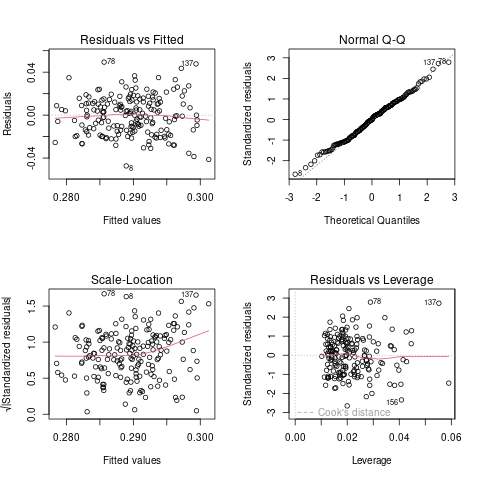

Supplement: Supporting Information — Additional supporting information can be found online in the Supporting Information section. Please find the MRI sequence parameters in the published file “supplemental.docx”. [file 5525213.f1.docx]
